# Serious complications associated with regadenoson administration for myocardial perfusion imaging

**DOI:** 10.1007/s12350-014-0048-5

**Published:** 2014-12-30

**Authors:** Therese Kitt, Jeanette Jiang

**Affiliations:** Astellas Scientific & Medical Affairs, Inc, 1 Astellas Way, Northbrook, IL 60062 USA

**To the Editor,**

The Editorial by Hage and Iskandrian^1^ regarding “Serious complications associated with regadenoson administration for myocardial perfusion imaging: A commentary” (September/October 2014 issue) includes a table as a guide for serious complications associated with regadenoson administration. In the table, the authors reported whether or not each listed event occurs with exercise and/or adenosine and that seizures have not been reported with adenosine. Astellas has received spontaneous post-marketing reports of seizures in patients administered adenosine, and the package insert for Adenoscan® was updated in 2005 to reflect these reports. The current package insert includes “seizure” in the Warnings section.^2^ In addition, there is one published case report of bradyasystole and seizures associated with the use of adenosine to terminate supraventricular tachycardia.^3^ Astellas would like to clarify that seizures can occur following the administration of adenosine, and prescribers should be aware of this risk and prepared to treat if necessary.^2^ The use of methylxanthines (e.g., caffeine, aminophylline, and theophylline) is not recommended in patients who experience seizures in association with adenosine and regadenoson.^2^
